# Development of interval-valued fuzzy GRA with SERVPERF based on subjective and objective weights for evaluation of airline service quality: A case study of Korea low-cost carriers

**DOI:** 10.1371/journal.pone.0219739

**Published:** 2019-08-06

**Authors:** Sanghoon Lee, Daekook Kang

**Affiliations:** 1 Department of Business Administration, College of Business & Economics, Hannam University, Daejeon, Republic of Korea; 2 Department of Industrial and Management Engineering, Inje University, Gimhae-si, Gyeongsangnam-do, Republic of Korea; National Taiwan University of Science and Technology, TAIWAN

## Abstract

As the airline industry has become ever-more competitive and profitability more tenuous, airline service quality management has grown more important to airlines. Although many studies have focused on the evaluation of airline service quality, some common limitations need to be noted. First, traditional fuzzy logics were utilized to present linguistic variables as fuzzy numbers. However, precise quantification of lower and upper bounds with a single number is often difficult; thus, interval-valued fuzzy sets that represent the lower and upper bounds in the fuzzy number as an interval form should be applied instead. Second, while some studies have applied various multiple-criteria decision-making method [MCDM] and the service quality (SERVQUAL) method for evaluation of airline service quality, few have utilized grey relational analysis (GRA, a simple and data-driven MCDM method applicable to environments with incomplete information) and the service performance (SERVPERF), a performance-based measure that can resolve the ambiguity issue of the expectations construct in SERVQUAL. Third, extant studies dealing with the issue of weighting criteria in the evaluation of airline service quality have focused only on either subjective or objective weights, though weighting criteria based on a combined objective/subjective approach would be much better than those just considering the subjective approach. The present study endeavored to fill these literature gaps by developing, for evaluation of airline service quality, interval-valued fuzzy GRA with SERVPERF based on both subjective and objective weights. It contributes to the field by incorporating the 22 criteria from SERVPERF to effectively account for the various characteristics of airline service. Additionally, it is the first study to utilize interval-valued fuzzy GRA together with a novel technique that combines a subjective/objective weighting method for integration of objective decision-matrix-derived information with subjective decision-maker preferences. The supplemental empirical case study of airline service evaluation, further, provides researchers and practitioners with a means of better understanding the proposed approach in the practical perspectives.

## Introduction

With the recent rapid growth in passenger traffic, airlines have been subjected to intense competition due to both the global economic downturn and passengers’ heightened awareness of service quality [1]. Under this circumstance, airlines have struggled to survive by strategies such as establishing more convenient routes, increasing the frequency of flights, and providing passengers with more promotional incentives [2, 3]. However, as the great majority of airlines have come to adopt these same or similar strategies, their marginal benefits and overall effectiveness have diminished. Thus, it has come to be recognized that in a highly competitive business environment, the provision, simply, of high-quality service is the core competitive advantage of an airline as well as the key to its profitability and sustained development [4]. This implies, further, that the monitoring and consequent improvement of service quality is the key to a successful airline.

A number of past studies have addressed issues pertinent to airline service quality evaluation using service quality (SERVQUAL) and multiple-criteria decision-making (MCDM). SERVQUAL is a multi-item instrument for measurement of service quality based on the gap model, in which service quality is a function of the difference between perception and expectation. [2] suggested a fuzzy weighted SERVQUAL model for evaluation of airline service quality. [1] applied SERVQUAL and the VIKOR method to improve the service qualities of domestic Taiwanese airlines. Among the various MCDM methods, some researchers have applied grey relational analysis (GRA) for evaluation of airline service quality because it is useful mathematically when dealing with a system with limited information. Indeed, GRA has strengths in uncertainty-intensive environments such as the airline service industry because the number of data sample that is necessary for the evaluation process is relatively smaller compared to other MCDM methods and information about the distribution of sample data is not needed. [5] applied fuzzy theory with factor analysis, analytic hierarchy process (AHP) and GRA to evaluate airline service quality. They used factor analysis to extract certain independent common-factors, fuzzy integral to integrate the performance ratings of inter-dependent attributes, AHP to determine the relative weights of attributes, and GRA to find the ranking of alternatives in terms of service quality. [6] applied a modified GRA method with SERVQUAL to improve service quality among domestic Taiwanese airlines.

Although all the aforementioned studies enlighten evaluation of airline service quality using GRA and SERVQUAL, some opportunities for improvement in practice need to be explored. First, in previous studies, fuzzy logics were utilized to address uncertain MCDM problems. Since airline service industry contains intangibility, perishability, inseparability and heterogeneity, it makes passengers more difficult for measuring airline service quality [3]. Specifically, service quality in airline industry encompasses a variety of activities that have uncertainty and vagueness that make it difficult to assess objective levels. For instance, there are various subjective evaluation criteria such as ticket reservation, purchasing, convenient ticketing process, service attitude of check-in attendant, comfortable and safe travelling, on-time departure and arrival and value-added services such as on-board services, seat comfort, and cleanliness, luggage transportation, customer complaint handling and lost baggage handling and services for delayed passengers [7]. In other words, perceptions of airline service quality depend on the linguistic aspects and preferences of different decision makers. Since the airline service evaluation problem includes various service quality dimensions and fuzzy logic that converts linguistic variables into evaluation scores, it is necessary to use fuzzy logic based MCDM method instead of probabilistic methods or machine learning methods. For this reason, many studies on the evaluation of airline service quality have utilized the fuzzy-based MCDM approach [2, 3, 8]. In fuzzy logics, linguistic variables are presented as fuzzy numbers that consist of lower and upper bounds. These lower and upper bounds in a fuzzy number are expressed as a single number. However, precise quantification of lower and upper bounds with a single number is often difficult. This is due to the fact that airline service tends to be evaluated with more qualitative and perceptional standards. Therefore, advanced fuzzy logic that includes qualitative and perceptual standards rather than existing fuzzy logic should be applied. With the recent development of interval-valued fuzzy sets (IVFSs), it is possible to represent the lower and upper bounds in a fuzzy number in an interval form. A fuzzy set is expressed in the form of membership functions. These membership functions are usually determined by experts, but they are ambiguous due to the subjectivity of the expert [7]. Therefore, various methods have been developed to generalize fuzzy sets to solve this problem. Among various methods, the IVFS is the most common generalization method of fuzzy set and it allocates an interval, not a single value, to upper and lower bounds in the membership function. As IVFSs can provide an additional degree of freedom to capture the uncertainty and the vagueness of the real world, they are more flexible in accounting for them. Therefore, the combination of IVFSs and MCDM methods offers impeccable utility in the field of airline service evaluation.

Second, although the previous studies have utilized SERVQUAL ([9], the most popular measure of airline service quality, it has been empirically demonstrated that service performance (SERVPERF), which uses customers’ perceived performance as a direct measure of service quality, is a more effective tool [10]. Specifically, because the SERVPERF instrument discards the expectation component and deals only with the performance component, it is free from problems related to how “expectation” is defined in SERVQUAL (i.e., as predicted value, ideal standard, or importance). All of this notwithstanding, the empirical research applying MCDM in tandem with the SERVPERF method for evaluation of airline service is relatively scarce.

Third, in the field of MCDM research focusing on the evaluation of airline service quality, scholars have begun to study how to effectively weight criteria to make decision-making more scientific. The approaches followed in previous studies can be divided into two categories: subjective weighting and objective weighting. The former, such as AHP, collects the subjective preferences of the decision makers. The majority of studies have utilized this approach to determine the weights of criteria. For instance, some studies have applied AHP [3, 11], while [8] collected the assessment results of decision makers from surveys and by averaging those scores, derived importance weights for criteria. The subjective weighting method can accurately reflect all decision makers’ different opinions on criteria weights. However, it is usually affected by decision makers’ wisdom, experience and information, all of which are difficult to define or describe exactly [12, 13]. Moreover, in subjective weighting, the greater the number of evaluating objects, the more difficult the evaluation work becomes [14]. On the other hand, the alternative weighting protocol, objective weighting (e.g., entropy weight), is based on data that are given in the decision matrix of the attributes for each alternative. The objective weighting approach can overcome the shortcomings of the subjective approach by eliminating man-made instabilities and yielding more realistic results [15, 16]. Among the research applying the objective weighting approach to the evaluation of airline service quality, [13] utilized entropy weight and grey relation analysis to evaluate the corporate social responsibility of airline services.

Still, extant studies dealing with the issue of weighting criteria in the evaluation of airline service quality also have some limitations. In the airline service industry, both subjective preferences of experts and objective assessment information are important. Because there are many subjective evaluation criteria causing uncertainty, the objective approach to the weighting of criteria is necessary in airline service evaluation [13]. Additionally, though, the examination of the importance weights of criteria also requires the professional knowledge of experts who are very familiar with airline service and its issues. Therefore, weighting criteria by a combined subjective/objective approach would be much better than by the subjective or objective approach alone. However, despite the glaring need for research data that might be suggestive of a novel combined subjective/objective approach, such information is still lacking.

Thus, motivated by limitations in previous studies, the present study undertook to fill that knowledge gap by developing an interval-valued fuzzy GRA with SERVPERF based on both subjective and objective weights for evaluation of airline service quality. First, in terms of deriving evaluation criteria, SERVPERF framework is utilized. Second, for deriving importance weights of criterion, integrated weight approach combining subjective and objective weights is applied. Especially, in this study, *averaging score method* is utilized as the subjective weighting approach for reflecting the group decision-making situation of the evaluation of airline service quality. Compared to AHP subjective weighting method, it has simple calculation process but effective. Also, Shannon entropy measure is used as the objective weighting for criteria. Lastly, GRA method is applied to evaluate the ranking of alternatives considering different importance weights of criteria derived from integrated weight approach.

This paper’s contribution is its suggested new MCDM model that reflects, and is practicably applicable to, the uniquely complex evaluation and decision-making environment of airline service. First, by utilizing interval-valued fuzzy sets, uncertainties in airline service evaluation can effectively be handled. Additionally, the SERVPERF scale is more efficient than the SERVQUAL scale, as it reduces by half the number of items to be measured [17]. Second, GRA is expected to be especially suitable for evaluation of airline service quality, as it is a simple, straightforward and flexible approach using different weighting coefficients for decision making circumstance with incomplete information [18, 19]. Compared to the other methods such as TOPSIS and VIKOR, GRA has strengths in the evaluation environment with multi-input, and data incompleteness [18]. While TOPSIS should consider both distance from the positive-ideal solution and the distance from the negative-ideal solution, GRA does not require these data. Instead, GRA only considers data difference between comparability sequence and reference sequence by measuring the degree of correlation between sequences [19]. Even though the calculation process of GRA is simple, it provides and precise and reliable results. Third, a novel weighting technique is proposed combining the subjective weighting method and the objective weighting method to integrate the subjective preferences of decision makers with decision-matrix-derived objective information.

As far as is known, this is the first attempt to combine interval-valued fuzzy sets, GRA, SERVPERF and an integrated subjective/objective weighting approach for evaluation of airline service quality. With this approach, airline service quality can be evaluated effectively by adjusting the reflection rate of subjective and objective weights according to different circumstances.

The rest of the paper is organized as follows. Section 2 introduces previous studies focusing on SERVPERF, interval-valued fuzzy sets, GRA, and subjective/objective weights. Section 3 explains the overall framework of this study and provides the detailed steps. Section 4 provides a case study to apply the proposed approach. In this section, sensitivity analysis is conducted to investigate the influence levels of subjective and objective weights. In addition, validation test for the proposed method is conducted in order to effectively demonstrate the methods improvement over current studies. Lastly, in section 5, the summarization and contribution of this study are explained and several limitations that anticipate future research are provided.

## Background

### SERVPERF

Monitoring of customer preferences is the most key factor determining the successful delivery of high-quality service [20, 21]. Correspondingly, service quality evaluation to capture the “voice of the customer” is indispensable to any improvement of service quality or enhancement of customer satisfaction [8]. For these purposes, various studies have been progressed in depth. Among many methods, SERVQUAL has been well used as the assessment method of service quality. SERVQUAL is a gap model in which service quality is a function of the difference between the perceptions and expectations of a service. When the SERVQUAL was firstly proposed, 10 main dimensions of service quality were considered as follows: *“(1) reliability; (2) responsiveness; (3) competence; (4) access; (5) courtesy; (6) communication; (7) credibility; (8) security; (9) understanding/knowing the customer; (10) tangibles”* [22]. However, in later work, these dimensions were reduced to three dimensions, namely *tangibles*, *reliability*, and *responsiveness*. In addition, *assurance* and *empathy* were added as new dimensions, so there were five main dimensions [23]. These dimensions contain 22 items for measurement of expectations and 22 corresponding items for measurement of perceptions. SERVQUAL has spawned a considerable amount of related follow-up research on its practical applications and theoretical dimensions. However, it has also been criticized from theoretical and operational perspectives [24]. [25] posited that, due to the ambiguity of the expectations construct, measurement of service quality as a difference or gap score is inappropriate for reflection of complex cognitive processes of service-quality perception. They insisted that one’s perception of service quality already entails the expectation of that service. Additionally, many researchers have insisted that a simple performance-based approach is a preferable means of measuring service quality [26]; [10, 27]. Motivated by mounting criticisms of SERVQUAL, [10] suggested SERVPERF, a performance-based measure of service quality. The SERVPERF instrument discards the expectation component and includes only 22 items for measurement of performance (P). It assumes that higher-perceived performance implies higher service quality [17]. Obviously, the SERVPERF scale is more efficient than that of the SERVQUAL, because it reduces by half the number of items to be measured [28]. [27] also determined that SERVPERF covers more of the variation in the global measure of service quality than can the SERVQUAL scale. SERVPERF consists of the five dimensions containing 22 sub-criteria as shown in Table 1: tangibles, reliability, responsiveness, assurance, and empathy.

**Table 1:** Definitions of five dimensions of SERVPERF.

Dimension	Definition	Number of items
Tangibles	Physical facilities, equipment and the appearance of the staff	4
Reliability	Ability for providing service dependably and accurately	5
Responsiveness	Ability to know and willingness to cater to customer needs	4
Assurance	Employees’ knowledge and courtesy and their ability for customer to inspire feeling of trust	4
Empathy	Ability of the staff to provide caring service to customers	5

### Interval-valued fuzzy sets

According to [29], it is very difficult to reasonably express situations that are complex, or difficult to define, using traditional quantification methods; thus the concept of linguistic variables, “which are variables whose values are words or sentences”, is needed [30–32]. To present this linguistic variable as a number in an interval [0,1], fuzzy sets theory has been frequently utilized. However, some studies have noted that the presentation of the linguistic variable in the form of traditional fuzzy sets is not adequate, because it is difficult to precisely quantify a decision maker’s opinion as a number in an interval [0,1] [33–35]. It is more appropriate, in fact, to represent the degree of certainty using interval value. To resolve this issue in ordinary fuzzy sets, interval-valued fuzzy numbers (IVFNs) have been defined and their operations suggested [36, 37]. In other words, considering the fact that, in some cases, determining precisely of this value is difficult, the membership value can be expressed as an interval, consisting real numbers [38]. This is the core concept of IVFNs. IVFSs have been widely utilized in the previous research such as approximate reasoning [39], performance evaluation [40], image filtering [41], and uncertainty measurement [42].

According to [37], the IVFS can be defined based on (−∞,∞) and is given by

$$A=\{\left(x,[\mu_{A}^{L}(x),\mu_{A}^{U}(x)]\right)\}$$ (1)

$$\mu_{A}^{L}(x),\mu_{A}^{U}(x):X\to [0,1]\;\forall x\in X\;\mu_{A}^{L}(x)\leq \mu_{A}^{U}(x),$$

$$\bar{\mu_{A}}(x)=[\mu_{A}^{L}(x),\mu_{A}^{U}(x)]$$

$$A=\{\left(x,\bar{\mu_{A}}(x)\right)\},x\in (-\infty ,\infty )$$

where $\mu_{A}^{L}(x)$ is the lower limit of the degree of membership and $\mu_{A}^{U}(x)$ is the upper limit.

**Fig 1:**
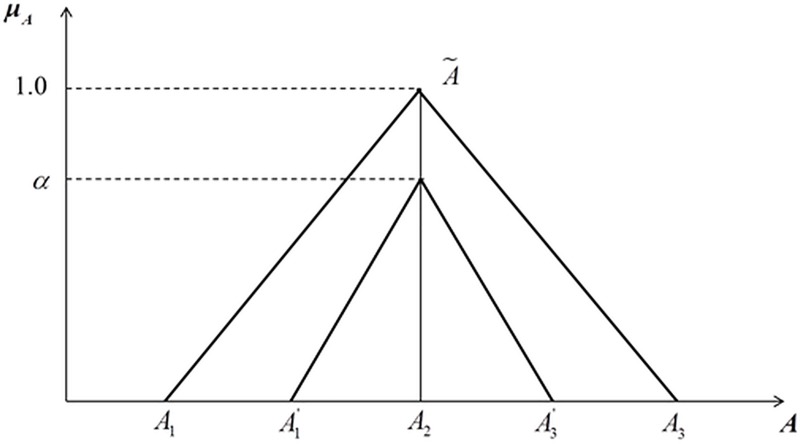
Membership functions of triangular interval-valued fuzzy number.

As illustrated in Fig 1, $\tilde{A}$ can be represented in triangular IVFNs.

$$\tilde{A}=\{\begin{array}{c} \left(A_{1},A_{2},A_{3}\right)\\ \left(A_{1}^{\prime },A_{2}^{\prime },A_{3}^{\prime }\right) \end{array}$$ (2)

To defuzzify interval-valued fuzzy numbers, distance measures can be utilized. There are various distance measures such as Euclidean distance, Mahalanobis distance, and cosine distance. The Euclidean distance is a formula for finding the shortest distance between two points in an n-dimensional space, using the Pythagorean theorem and corresponds to the most intuitive and general concept of distance [43]. Mahalanobis distance measures distance relative to the centroid—a base or central point which can be thought of as an overall mean for multivariate data. It measures distance by considering the covariance of two vectors. The cosine distance measures the angle difference rather than the distance between the two coordinates. If these two vectors are orthogonal, this measure is 0, and if they are in the same direction, it is 1.

Different distance measures can affect the final result of the evaluation. However, among various distance measures, the Euclidean distance measure should be applied for measuring the distance between IFVNs because in interval-valued fuzzy numbers, it is important to measure the absolute distance rather than the distance between the centroid and the two points (Mahalanobis distance) and the angle difference between two points (cosine distance). Therefore, in this study, we use the Euclidean distance measure for defuzzifying interval-valued fuzzy numbers.

Given two IVFNs $\tilde{A}=[(A_{1},A_{1}^{'});A_{2};(A_{3},A_{3}^{'})]$ $\tilde{B}=[(B_{1},B_{1}^{'});B_{2};(B_{3},B_{3}^{'})]$, according to [38, 44, 45], we have:

$$\mathrm{Definition}\;1.\;\mathrm{If}\;•\in (+,-,\times ,÷),\;\mathrm{then}\;\tilde{A}•\tilde{B}=[(A_{1}•B_{1},A_{1}^{'}•B_{1}^{'});A_{2}•B_{2};(A_{3}•B_{3},A_{3}^{'}•B_{3}^{'})]$$ (3)

**Definition 2.** The normalized Euclidean distance between $\tilde{A}$ and $\tilde{B}$ is as follows:

$$D(\tilde{A},\tilde{B})=\sqrt{\frac{1}{6}\sum \left[{\left(A_{1}-B_{1}\right)}^{2}+{\left(A_{1}^{'}-B_{1}^{'}\right)}^{2}+2\times {\left(A_{2}-B_{2}\right)}^{2}+{\left(A_{3}-B_{3}\right)}^{2}+{\left(A_{3}^{'}-B_{3}^{'}\right)}^{2}\right]}$$ (4)

When $\tilde{B}=[(0,0);0;(0,0)]$, we can defuzzify fuzzy number $\tilde{A}$ in the form of a specific crisp value based on Eq (4).

Here, we use a linguistic variable as a rating to measure the performance value of the best alternative plan according to the following five basic linguistic terms [38]: “very good (VG),” “good (G),” “moderately good (G),” “fair (F),” “moderately poor (MP),” “poor (P),” and “very poor (VP).” This study used the IVFNs as shown in Table 2.

**Table 2:** IVFNs of linguistic variables for rating of alternatives.

Linguistic variables	IVFNs
Very poor (VP)	[(0,0);0;(1,1.5)]
Poor (P)	[(0,0.5);1;(2.5,3.5)]
Moderately Poor (MP)	[(0,1.5);3;(4.5,5.5)]
Fair (F)	[(2.5,3.5),5,(6.5,7.5)]
Moderately Good (MG)	[(4.5,5.5),7,(8,9.5)]
Good (G)	[(5.5,7.5),9,(9.5,10)]
Very good (VG)	[(8.5,9.5),10,(10,10)]

Linguistic variables are used to measure the importance weight of each criterion [38], as “very high (VH),” “High (H),” “medium high (MH),” “medium (M),” “medium low (ML),” “low (L),” and “very Low (VL).” The IVFNs used in this study are listed in Table 3.

**Table 3:** IVFNs of linguistic variables for importance weights of criteria.

Linguistic variables	IVFNs
Very low (VL)	[(0,0);0;(0.1,0.15)]
Low (L)	[(0,0.05);0.1;(0.25,0.35)]
Medium low (ML)	[(0,0.15);0.3;(0.45,0.55)]
Medium (M)	[(0.25,0.35),0.5,(0.65,0.75)]
Medium high (MH)	[(0.45,0.55),0.7,(0.8,0.95)]
High (H)	[(0.55,0.75),0.9,(0.95,1)]
Very high (VH)	[(0.85,0.95),1,(1,1)]

### Grey relational analysis

Grey theory was suggested by [46] as a means of investigating the degree of relation among various attributes in an MCDM problem. It is a theory being applied for the decision making in the systems or problems that are “grey”. Here, “Grey” means that some information is known and other information is unknown [47]. It is mathematically useful when dealing with an environment with limited information. As a part of grey theory, grey relational analysis (GRA) is one of MCDM methods, which concerns the complicated relationships between multiple criteria [18, 19, 48]. Compared with the other, conventional methods, which require large amounts of data, GRA possesses the following advantages [19, 46, 49]: (1) it follows a simple and easy calculation process; (2) the amount of sample data necessary for the calculation is small; (3) a typical distribution of sample data is not required; (4) the quantified outcomes from the grey relational grade do not result in conclusions that are contradictory to the qualitative analysis; (5) it provides the flexibility that enables imposition of different weighting coefficients on factors. Owing to these advantages, GRA has been widely used in practice for various decision-making problems [6, 19, 50–54].

The analytic procedure of GRA normally is comprised of four stages [18, 55]: grey relational generating, standard series setting, calculation of the grey relational coefficient, and calculation of the grey relational grade. The main procedure of GRA is to translate the alternative rating into a comparability sequence. This is the grey relational generating. Then, in the stage of standard series setting, a reference sequence (ideal sequence) is derived. Then, the grey relational coefficient is calculated by analyzing the relation degree between comparability sequence and the reference sequence. Lastly, by using grey relational coefficients, the grey relational grade is calculated. If a comparability sequence for alternative has the highest grey relational grade with the reference sequence, this alternative can be the best among all alternatives. The detailed GRA procedures will be explained in Section 3.

### Subjective and objective weights

In MCDM problems, assessing the weights of criteria is an important issue. Weights of criteria should reflect the respective relative importance in the decision-making process [56]. Because the evaluation of weights of criteria involves diverse opinions and meanings, we cannot assume that each evaluation criterion has equal importance [57]. There are two categories of weighting approach: the subjective approach and objective approach. First, in the subjective approach, the weights of criteria are determined solely based on the preferences or judgments of decision makers. There are various subjective methods such as *AHP*, which calculates the weights of criteria based on the pairwise comparisons of the criteria, and *averaging score method*, which is appropriate for group decision-making situations wherein pairwise comparisons are not needed. In this study, the *averaging score method* could be utilized as the subjective weighting approach for reflecting the group decision-making situation of the evaluation of airline service quality.

By contrast, the objective approach determines the weights of criteria by applying mathematical approach automatically without any consideration of the preferences of decision makers. Especially, entropy weighting can be recognized as the representative objective weighting approach. The entropy concept, which is a measure of information uncertainty, was firstly proposed by Shannon and Weaver (1947). As is known, in the field of thermodynamics, entropy is the measure of the disorder in a system. Having been transferred from the field of thermodynamics to the information domain, Shannon entropy can be widely employed to evaluate the degree of disorder and the effectiveness of the information for a system [13].

Shannon suggested the *H* measure, which satisfies the following three properties for all *p*_*i*_ within the estimated joint probability distribution P [58]:

1. *H* is a continuous positive function;
2. If all *p*_*i*_ are equal, $p_{i}=\frac{1}{n}$, *H* should be a monotonically increasing function of *n*
3. For all *n*≥2, $H(p_{1},p_{2},\ldots ,p_{n})=H(p_{1}+p_{2},p_{3},\ldots ,p_{n})+(p_{1}+p_{2})H(\frac{p_{1}}{p_{1}+p_{2}},\frac{p_{2}}{p_{1}+p_{2}})$.

Shannon showed that the only function that satisfies these properties is

$$H(P)=-\sum_{i}p_{i}\log(p_{i})$$ (5)

This concept of Shannon’s has been well deployed as a weighting calculation method [16, 56]. In entropy measure, the smaller the entropy value, the smaller the degree of disorder in the system and the higher the weight [59]. In other words, the higher the value of the entropy, the smaller the entropy weight, and so also the smaller the different alternatives in the specific criterion, the less information the specific criterion provides, and the less important this criterion becomes in the evaluation process [56]. In this paper, we utilize the entropy measure as an objective weight.

According to the above-outlined literature, we can make sure that integrated weights based on subjective weight and entropy measure with GRA are workable when dealing with the evaluation problem of airline service quality. Having briefly reviewed GRA, subjective weight, and entropy, we propose their operation procedures entailing integrated weights and GRA as a means of evaluating airline service quality under our study. The detailed calculation steps will be explained in Section 3.

## Proposed approach for evaluation of airline service quality

### Overall framework of the proposed approach

**Fig 2:**
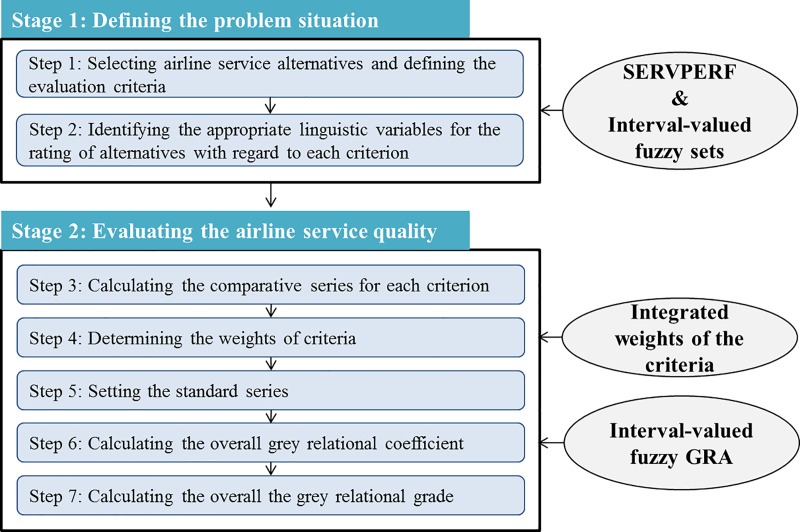
Overall framework.

The overall framework of the proposed approach for evaluation of airline service quality is illustrated in Fig 2. As indicated, the process consists of two main stages: *defining the problem situation*, and *evaluating the airline service quality*; they together include, as detailed procedures, a total of 7 sub-steps.

### Defining the problem situation

#### Step 1. Selecting airline service alternatives and defining evaluation criteria

As the first step in the evaluation of airline service quality, several airline service alternatives are selected as evaluation alternatives. In this step, airline service alternatives that generally belong to the same category such as low-cost airlines or full-service carriers, and provide similar flight routes, are very important, because the significant criteria that customers consider do no highly differ among airline service alternatives that belong to the same category. Thus, from the same airline service category, several airline service alternatives can be selected as evaluation alternatives. Then, for the selected airline service alternatives, the evaluation criteria including the five dimensions and sub-criteria of SERVPERF, proposed by [9] and [10], are utilized. Additionally, for the present study, we also adopted airline-service-focused 22 sub-criteria from previous studies [28, 60] and slightly change the wording to suit the current research.

#### Step 2. Identifying appropriate linguistic variables for rating of alternatives

Here, a decision matrix (***Z***) is established to obtain assessment scores for airline services. Consider a two-layer situation of ***m*** alternatives ***A*** = {***A***_1_,***A***_2_,…,***A***_***m***_} that include ***n*** criteria ***C*** = {***C***_1_,***C***_2_,…,***C***_***n***_}. Then, a decision group consisting of ***k*** experts evaluates alternatives with linguistic variables

$$\begin{array}{l} \;C_{1}\;C_{2}\;C_{n}\\ {\tilde{Z}}^{p}=\begin{array}{c} A_{1}\\ A_{2}\\ \ldots \\ A_{m} \end{array}\left[\begin{array}{cccc} {\tilde{z}}_{11}^{p} & {\tilde{z}}_{12}^{p} & \ldots & {\tilde{z}}_{1n}^{p}\\ {\tilde{z}}_{21}^{p} & {\tilde{z}}_{22}^{p} & \ldots & {\tilde{z}}_{2n}^{p}\\ \ldots & \ldots & \ldots & \ldots \\ {\tilde{z}}_{m1}^{p} & {\tilde{z}}_{m2}^{p} & \ldots & {\tilde{z}}_{mn}^{p} \end{array}\right] \end{array}$$ (6)

where ${\tilde{z}}_{ij}^{p}=[(a_{ij}^{p},a^{'}{}_{ij}^{p}),b_{ij}^{p},(c_{ij}^{p},c^{'}{}_{ij}^{p})]$ is an evaluation rating of the ***p***th expert in a decision group consisting of ***k*** experts with triangular IVFNs.

Then, in a group decision environment with ***k*** experts, the aggregated decision matrix for the alternative rating in terms of each criterion is derived as

$${\tilde{y}}_{ij}=\frac{1}{K}[{\tilde{z}}_{ij}^{1}+{\tilde{z}}_{ij}^{2}+\ldots +{\tilde{z}}_{ij}^{k}]$$ (7)

$$\begin{array}{l} \;C_{1}\;C_{2}\;C_{n}\\ \tilde{Y}=\frac{1}{k}\sum_{p=1}^{k}{\tilde{Z}}^{p}=\begin{array}{c} A_{1}\\ A_{2}\\ \ldots \\ A_{m} \end{array}\left[\begin{array}{cccc} {\tilde{y}}_{11} & {\tilde{y}}_{12} & \ldots & {\tilde{y}}_{1n}\\ {\tilde{y}}_{21} & {\tilde{y}}_{22} & \ldots & {\tilde{y}}_{2n}\\ \ldots & \ldots & \ldots & \ldots \\ {\tilde{y}}_{m1} & {\tilde{y}}_{m2} & \ldots & {\tilde{y}}_{mn} \end{array}\right]. \end{array}$$ (8)

In this step, all of the evaluation rating for every alternative are processed into a comparability sequence. When the performance units for each criterion are different, the impact of some criteria may be neglected. Also, if the goals of these criteria are different, it may cause inaccurate results in the evaluation process [61]. Thus, processing analogous to normalization is necessary. This is the grey relational generating. Here, before we calculate the comparative series for each criterion, in this stage, *IVFNs of the aggregated decision matrix for the rating of alternatives* should be defuzzified as a crisp value based on Eq (4), as

$$\begin{array}{l} \;C_{1}\;C_{2}\;C_{n}\\ Y=\mathrm{defuzz}(\tilde{Y})=\begin{array}{c} A_{1}\\ A_{2}\\ \ldots \\ A_{m} \end{array}\left[\begin{array}{cccc} \mathrm{defuzz}({\tilde{y}}_{11}) & \mathrm{defuzz}({\tilde{y}}_{12}) & \ldots & \mathrm{defuzz}({\tilde{y}}_{1n})\\ \mathrm{defuzz}({\tilde{y}}_{21}) & \mathrm{defuzz}({\tilde{y}}_{22}) & \ldots & \mathrm{defuzz}({\tilde{y}}_{2n})\\ \ldots & \ldots & \ldots & \ldots \\ \mathrm{defuzz}({\tilde{y}}_{m1}) & \mathrm{defuzz}({\tilde{y}}_{m2}) & \ldots & \mathrm{defuzz}({\tilde{y}}_{mn}) \end{array}\right] \end{array}$$ (9)

$$\mathrm{defuzz}({\tilde{y}}_{ij})=D({\tilde{y}}_{ij},0)=\sqrt{\frac{1}{6}\sum \left[{\left(a_{ij}-0\right)}^{2}+{\left(a_{ij}^{'}-0\right)}^{2}+2\times {\left(b_{ij}-0\right)}^{2}+{\left(c_{ij}-0\right)}^{2}+{\left(c_{ij}^{'}-0\right)}^{2}\right]}$$ (10)

where $\mathrm{defuzz}({\tilde{y}}_{ij})$ is the defuzzified value of the aggregated decision matrix for the rating of alternatives, and ${\tilde{y}}_{ij}=[(a_{ij},a_{ij}^{'}),b_{ij},(c_{ij},c_{ij}^{'})]$.

### Evaluating airline service quality

#### Step 3: Calculating the comparative series for each criterion

If there are ***m*** alternatives and ***n*** criteria, the ***i***th alternative can be expressed as ***Y***_***i***_ = (***y***_***i***1_,***y***_***i***2_,…,***y***_***ij***_,…,***y***_***in***_), where ***y***_***ij***_ is the defuzzified performance value of criteria ***j*** of the airline service of alternative ***i***. The term ***Y***_***i***_ can be translated into the comparability sequence ***X***_***i***_ = (***x***_***i***1_,***x***_***i***2_,…,***x***_***ij***_,…,***x***_***in***_) as

$$X_{ij}=\frac{y_{ij}-Min\{y_{ij},i=1,2,\ldots ,m\}}{Max\{y_{ij},i=1,2,\ldots ,m\}-Min\{y_{ij},i=1,2,\ldots ,m\}}\;\mathrm{for}\;i=1,2,\ldots ,m\;j=1,2,\ldots ,n.$$ (11)

#### Step 4. Determining the weights of criteria

First, subjective weights of criteria based on the averaging score method are derived in this step. In a group decision environment with ***k*** experts, the aggregated decision matrix for the importance weight of each criterion can be calculated using the *averaging score method* as

$${\tilde{W}}^{p}=\begin{array}{cc} \begin{array}{c} C_{1}\\ C_{2}\\ \vdots \\ C_{j}\\ \vdots \\ C_{n} \end{array} & \left[\begin{array}{c} {\tilde{w}}_{1}^{p}\\ {\tilde{w}}_{2}^{p}\\ \vdots \\ {\tilde{w}}_{j}^{p}\\ \vdots \\ {\tilde{w}}_{n}^{p} \end{array}\right] \end{array}$$ (12)

where ${\tilde{w}}_{j}^{p}=[(d_{j}^{p},d^{'}{}_{j}^{p}),e_{j}^{p},(f_{j}^{p},f^{'}{}_{j}^{p})]$ is the importance weight of the ***p***th expert in a decision group consisting of ***k*** experts with triangular IVFNs,

$${\tilde{w}}_{j}=\frac{1}{K}[{\tilde{w}}_{j}^{1}+{\tilde{w}}_{j}^{2}+\ldots +{\tilde{w}}_{j}^{k}]\;\mathrm{for}\;j=1,2,\ldots ,n$$ (13)

$$\tilde{W}=\frac{1}{k}\sum_{p=1}^{k}{\tilde{W}}^{p}=\begin{array}{cc} \begin{array}{c} C_{1}\\ C_{2}\\ \vdots \\ C_{j}\\ \vdots \\ C_{n} \end{array} & [\begin{array}{c} {\tilde{w}}_{1}\\ {\tilde{w}}_{2}\\ \vdots \\ {\tilde{w}}_{j}\\ \vdots \\ {\tilde{w}}_{n} \end{array}]. \end{array}$$ (14)

Eq (13) shows the averaging score method for aggregation of the assessment results of experts, and Eq (14) represents the average values of $\tilde{W}$ denoted by the experts. Here, “+” is the sum operator as shown in Definition 1. Then, based on the values of $\tilde{W}$, the defuzzified subjective weights of the criteria can be derived using Eq (4) as

$$W^{Sub}=\mathrm{defuzz}(\tilde{W})=\begin{array}{cc} \begin{array}{c} C_{1}\\ C_{2}\\ \vdots \\ C_{j}\\ \vdots \\ C_{n} \end{array} & \left[\begin{array}{c} \mathrm{defuzz}({\tilde{w}}_{1})\\ \mathrm{defuzz}({\tilde{w}}_{2})\\ \vdots \\ \mathrm{defuzz}({\tilde{w}}_{j})\\ \vdots \\ \mathrm{defuzz}({\tilde{w}}_{n}) \end{array}\right] \end{array}$$ (15)

$$\mathrm{defuzz}({\tilde{w}}_{j})=D({\tilde{w}}_{j},0)=\sqrt{\frac{1}{6}\sum \left[{\left(d_{j}-0\right)}^{2}+(d_{j}^{'}-0{)}^{2}+2\times {\left(e_{j}-0\right)}^{2}+{\left(f_{j}-0\right)}^{2}+{\left(f_{j}^{'}-0\right)}^{2}\right]}$$ (16)

where $\mathrm{defuzz}({\tilde{w}}_{j})$ is the defuzzified value of the subjective weights of the criteria and ${\tilde{w}}_{j}=[(d_{j},d_{j}^{'}),e_{j},(f_{j},f_{j}^{'})]$.

Second, objective weights of criteria using entropy measure are derived. In order to determine objective weights using the entropy measure, the decision matrix is normalized based on $Y=\mathrm{defuzz}(\tilde{Y})$ as

$$p_{ij}=\frac{\mathrm{defuzz}({\tilde{y}}_{ij})}{\sum_{i=1}^{m}\mathrm{defuzz}({\tilde{y}}_{ij})}.$$ (17)

After deriving the normalized decision matrix, we can calculate the entropy values *e*_*j*_ as

$$e_{j}=-k\sum_{j=1}^{n}p_{ij}\ln p_{ij}$$ (18)

where *k* is Boltzman’s constant, which equals *k* = (ln(*m*))^−1^.

The degree of diversification *div*_*i*_ of the intrinsic information of each criterion *C*_*j*_(*j* = 1,2,…,*n*) can be calculated as

$$div_{j}=1-e_{j}.$$ (19)

The value *div*_*j*_ represents the inherent contrast intensity of *C*_*j*_. Thus, the higher the *div*_*j*_ is, the more important the criterion *C*_*j*_ is to the problem. Finally, the objective weight for each criterion can be obtained as

$$W_{j}^{Obj}=\frac{div_{j}}{\sum_{j}div_{j}}.$$ (20)

Lastly, integrated weights of criteria are calculated. In consideration of both the objective and subjective weights, the integrated weights of the criteria are calculated as

$$W_{j}^{Integ}=\alpha W_{j}^{Sub}+(1-\alpha )W_{j}^{Obj}$$ (21)

where $W_{j}^{Integ}$ is the integrated weight of the *j*th criterion, and *α* and 1−*α* are coefficient values between 0 and 1 denoting the subjective and objective weights respectively.

#### Step 5: Setting the standard series (reference sequence)

After the grey relational generating procedure using Eq (11), all values are convert to [0,1]. For a criteria ***j*** of an airline service of alternative ***i***, if the value ***x***_***ij***_ is equal to 1, or closer to 1 than the values of other alternatives, it means that the performance of alternative ***i*** is the best for the criteria ***j***. Thus, an alternative can be the best if all of its performance values are nearest to or equal to 1. However, usually, this alternative does not exist. This paper then sets the reference sequence ***X***_0_ as (***x***_01_,***x***_02_,…,***x***_0***j***_,…,***x***_0***n***_) = (1,1,…,1,…,1).

#### Step 6: Calculating the overall grey relational coefficient

The grey relational coefficient is utilized to determine how close ***x***_***ij***_ is to ***x***_0***j***_. The larger the grey relational coefficient, the closer ***x***_***ij***_ and ***x***_0***j***_ are. The grey relational coefficient can be calculated as

$$\gamma (x_{0j},x_{ij})=\frac{\Delta_{\min}+\zeta \Delta_{\max}}{\Delta_{ij}+\zeta \Delta_{\max}}\;(\zeta :\;\mathrm{the}\;\mathrm{distinguishing}\;\mathrm{coefficient},\;\zeta \in (0,1))$$ (22)

where ***i*** = 1,2,…,***m j*** = 1,2,…,***n***.

In Eq (22), ***γ***(***x***_0***j***_,***x***_***ij***_) is the grey relational coefficient between ***x***_***ij***_ and ***x***_0***j***_, and

$$\begin{array}{l} \Delta_{ij}=\left|x_{0j}-x_{ij}\right|,\\ \Delta_{\min}=Min\{\Delta_{ij},i=1,2,\ldots ,m;j=1,2,\ldots ,n\},\\ \Delta_{\max}=Max\{\Delta_{ij},i=1,2,\ldots ,m;j=1,2,\ldots ,n\}. \end{array}$$ (23)

Here, the distinguishing coefficient is used to expand the range of the grey relational coefficient. The differences among the grey relational coefficients for the respective alternatives will always change when different distinguishing coefficients are adopted; but, no matter what the distinguishing coefficient is, the ranking of alternatives is always the same [18]. In this paper, the distinguishing coefficient is set as 0.5, which is the generally set value in previous studies, while some other, different distinguishing coefficients are tested for analysis.

#### Step 7: Calculating the overall grey relational grade

In this step, the grey relational grade can be calculated using the weighting coefficients of the decision factors according to the formulation

$$\Gamma (X_{0},X_{i})=\sum_{j=1}^{n}W_{j}^{Integ}\gamma (x_{0j},x_{ij})\;\mathrm{for}\;i=1,2,\ldots ,m$$ (24)

where $W_{j}^{Integ}$ is the integrated weight of the *j*th criterion and Γ(***X***_0_,***X***_***i***_) is the grey relational grade between ***X***_***i***_ and ***X***_0_.

This shows the degree of relation between the reference sequence and the comparability sequence. As noted above, on each criterion, the reference sequence is the best performance that could be achieved by any among all comparability sequences. Therefore, if a comparability sequence for an alternative is awarded the highest grey relational grade, it implies that the comparability sequence is most close to the reference sequence, thus, the alternative would be the best choice.

## Empirical case study

To show the effectiveness of the proposed approach, the case study is suggested. In post-deregulation South Korea, traffic has grown and competition among airlines has increased. Especially, low-cost carriers (LCCs) emerged following the rapid growth of Korean tourism beginning in 2005, and the competition among them has been fierce. In this environment, South Korean LCCs are struggling to survive by providing service quality equal to that of full-service carriers (FSCs) while offering lower fares as a strategic tool. In fact, South Korean LCCs use the same low-fare-based strategies to satisfy customers and to encourage their repeat business [62]. However, the marginal benefit and effectiveness of these strategies have gradually declined. Thus, the quality of an LCC is a more vital factor than is a low fare, since quality is the key attractant of passengers. Thus, the case study focused on the evaluation of airline service quality, especially LCC service quality.

### Defining the problem situation

#### Step 1. Selecting airline service alternatives and defining evaluation criteria

As the first step, five widely used LCCs in South Korea were selected as the alternatives: *Airline service 1 (****A***_1_*)*, *Airline service 2 (****A***_2_*)*, *Airline service 3 (****A***_3_*)*, *Airline service 4 (****A***_4_*)*, and *Airline service 5 (****A***_5_*)*. For these alternatives, evaluation criteria of the five dimensions and sub-criteria of SERVPERF were selected by re-constructing the scheme of previous studies, as shown in the following Table 4 [9, 10, 28, 60].

**Table 4:** Evaluation criteria of five dimensions and sub-criteria of SERVPERF.

Tangibles	
***C***_1_	Up-to-date equipment & technology
***C***_2_	Comfort and cleanliness of seat
*C*_3_	Appearance of the physical facilities of this airline
***C***_4_	Appearance of flight attendants
Responsiveness	
*C*_5_	Courtesy of flight attendants
*C*_6_	Handling of delays
*C*_7_	Flight attendants’ speed handling requests
*C*_8_	Flight attendants’ approach to unexpected situations
*C*_9_	Flight attendants’ willingness to help
Reliability	
*C*_10_	Flight Safety
*C*_11_	On-time departure and arrival
*C*_12_	Truly providing committed services
*C*_13_	Consistent ground/in-flight services
Assurance	
*C*_14_	Professional training of flight attendants
*C*_15_	Service attitude of check-in attendant (ticket reservations and sales)
*C*_16_	Accuracy of various operations
*C*_17_	Flight attendants’ knowledge in answering questions
Empathy	
***C***_**18**_	Flight attendants’ behavior toward delayed passengers
***C***_**19**_	Individual attention to passengers
***C***_**20**_	Understanding of passengers’ specific needs
***C***_**21**_	Convenient ticketing process
***C***_**22**_	Customer complaint handling

For the second step, a group comprised of 3 decision makers (***DM***_1_,***DM***_2_,***DM***_3_) evaluated the service quality with linguistic expressions for the sub-criteria and alternatives. Here, we selected 3 experts on Korea LCC industry. They specialized in field of service quality management. Moreover, these experts are heavy users of Korea LCCs, so they have extensive information for evaluating service quality of Korea LCCs. The criteria and alternatives were evaluated on the linguistic scale as shown in Table 5.

**Table 5:** Linguistic variables for rating of alternatives.

	Evaluation scores assessed by 3 decision makers (*DM*_1_,*DM*_2_,*DM*_3_)					
		*A*_1_	*A*_2_	*A*_3_	*A*_4_	*A*_5_
Tangibles	***C***_**1**_	G,MG,VG	G,G,MG	G,VG,MG	G,MG,MG	MG,G,G
	***C***_**2**_	F,F,G	G,F,F	F,MP,G	F,MP,F	MP,F,P
	***C***_**3**_	F,G,G	VG,G,G	G,G,F	F,F,G	G,G,F
	***C***_**4**_	G,MG,VG	G,VG,MG	G,G,VG	F,G,MG	F,VG,G
Responsiveness	***C***_**5**_	G,VG,VG	VG,VG,G	G,G,G	MG,G,VG	MG,G,VG
	***C***_**6**_	G,VG,VG	VG,G,G	G,VG,G	MG,G,G	G,G,G
	***C***_**7**_	VG,G,G	G,VG,G	MG,G,G	VG,G,G	G,G,VG
	***C***_**8**_	MG,G,G	VG,G,MG	G,F,MG	F,MG,MG	G,G,MG
	***C***_**9**_	F,G,MG	F,MP,MG	G,F,MG	G,G,VG	VG,G,MG
Reliability	***C***_**10**_	MG,G,G	G,MG,VG	F,MG,G	G,MG,F	G,MG,G
	***C***_**11**_	G,MG,F	G,G,VG	F,G,VG	MG,VG,G	G,G,F
	***C***_**12**_	G,VG,G	G,F,MG	G,F,F	G,G,MG	G,F,MG
	***C***_**13**_	VG,G,G	G,G,VG	MG,G,VG	MG,MG,G	F,G,G
Assurance	***C***_**14**_	G,VG,VG	VG,G,G	G,G,MG	G,VG,G	VG,VG,G
	***C***_**15**_	G,G,MG	F,MG,G	VG,G,G	G,G,VG	G,G,VG
	***C***_**16**_	G,VG,G	G,VG,VG	VG,VG,G	G,VG,MG	MG,MG,G
	***C***_**17**_	F,G,G	MG,MG,F	F,MG,G	G,G,MG	MG,F,G
Empathy	***C***_**18**_	F,MG,MP	MP,MP,F	MP,MP,MP	F,MP,F	MG,MG,F
	***C***_**19**_	MG,MG,F	F,MG,MP	F,F,G	MG,MG,F	F,MG,G
	***C***_**20**_	F,MG,MG	MG,F,MP	G,MG,F	MP,G,MG	MG,F,F
	***C***_**21**_	MG,MG,G	G,MG,MG	F,MG,G	MG,F,F	MG,G,MG
	***C***_**22**_	MG,MG,G	F,MG,MG	G,G,MG	MG,F,F	G,G,G

The evaluation results were aggregated as shown in Table 6. According to Eqs (7) and (8), we could easily obtain the aggregated decision matrix for alternative rating regarding each criterion. For instance, the aggregated fuzzy value ${\tilde{y}}_{11}$ could be derived as

G=[(5.5,7.5),9,(9.5,10)],MG=[(4.5,5.5),7,(8,9.5)],VG=[(8.5,9.5),10,(10,10)],

$$\begin{array}{l} {\tilde{y}}_{11}=[(\frac{5.5+4.5+8.5}{3},\frac{7.5+5.5+9.5}{3}),\frac{9+7+10}{3},(\frac{9.5+8+9.5}{3},\frac{10+9.5+10}{3})]\\ =[(6.2,7.5),8.7,(9.2,9.8)]. \end{array}$$

**Table 6:** IFVNs of aggregated decision matrix for rating of alternatives.

	*A*_1_	*A*_2_	*A*_3_	*A*_4_	*A*_5_
***C***_**1**_	[(6.2,7.5),8.7,(9.2,9.8)]	[(5.2,6.8),8.3,(9.0,9.8)]	[(6.2,7.5),8.7,(9.2,9.8)]	[(4.8,6.2),7.7,(8.5,9.7)]	[(5.2,6.8),8.3,(9.0,9.8)]
***C***_**2**_	[(3.5,4.8),6.3,(7.5,8.3)]	[(3.5,4.8),6.3,(7.5,8.3)]	[(2.7,4.2),5.7,(6.8,7.7)]	[(1.7,2.8),4.3,(5.8,6.8)]	[(0.8,1.7),3.0,(4.5,5.5)]
***C***_**3**_	[(4.5,6.2),7.7,(8.5,9.2)]	[(6.5,8.2),9.3,(9.7,10.0)]	[(4.5,6.2),7.7,(8.5,9.2)]	[(3.5,4.8),6.3,(7.5,8.3)]	[(4.5,6.2),7.7,(8.5,9.2)]
***C***_**4**_	[(6.2,7.5),8.7,(9.2,9.8)]	[(6.2,7.5),8.7,(9.2,9.8)]	[(6.5,8.2),9.3,(9.7,10.0)]	[(4.2,5.5),7.0,(8.0,9.0)]	[(5.5,6.8),8.0,(8.7,9.2)]
***C***_**5**_	[(7.5,8.8),9.7,(9.8,10.0)]	[(7.5,8.8),9.7,(9.8,10.0)]	[(5.5,7.5),9.0,(9.5,10.0)]	[(6.2,7.5),8.7,(9.2,9.8)]	[(6.2,7.5),8.7,(9.2,9.8)]
***C***_**6**_	[(7.5,8.8),9.7,(9.8,10.0)]	[(6.5,8.2),9.3,(9.7,10.0)]	[(6.5,8.2),9.3,(9.7,10.0)]	[(5.2,6.8),8.3,(9.0,9.8)]	[(5.5,7.5),9.0,(9.5,10.0)]
***C***_**7**_	[(6.5,8.2),9.3,(9.7,10.0)]	[(6.5,8.2),9.3,(9.7,10.0)]	[(5.2,6.8),8.3,(9.0,9.8)]	[(6.5,8.2),9.3,(9.7,10.0)]	[(6.5,8.2),9.3,(9.7,10.0)]
***C***_**8**_	[(5.2,6.8),8.3,(9.0,9.8)]	[(6.2,7.5),8.7,(9.2,9.8)]	[(4.2,5.5),7.0,(8.0,9.0)]	[(3.8,4.8),6.3,(7.5,8.8)]	[(5.2,6.8),8.3,(9.0,9.8)]
***C***_**9**_	[(4.2,5.5),7.0,(8.0,9.0)]	[(2.3,3.5),5.0,(6.3,7.5)]	[(4.2,5.5),7.0,(8.0,9.0)]	[(6.5,8.2),9.3,(9.7,10.0)]	[(6.2,7.5),8.7,(9.2,9.8)]
***C***_**10**_	[(5.2,6.8),8.3,(9.0,9.8)]	[(6.2,7.5),8.7,(9.2,9.8)]	[(4.2,5.5),7.0,(8.0,9.0)]	[(4.2,5.5),7.0,(8.0,9.0)]	[(5.2,6.8),8.3,(9.0,9.8)]
***C***_**11**_	[(4.2,5.5),7.0,(8.0,9.0)]	[(6.5,8.2),9.3,(9.7,10.0)]	[(5.5,6.8),8.0,(8.7,9.2)]	[(6.2,7.5),8.7,(9.2,9.8)]	[(4.5,6.2),7.7,(8.5,9.2)]
***C***_**12**_	[(6.5,8.2),9.3,(9.7,10.0)]	[(4.2,5.5),7.0,(8.0,9.0)]	[(3.5,4.8),6.3,(7.5,8.3)]	[(5.2,6.8),8.3,(9.0,9.8)]	[(4.2,5.5),7.0,(8.0,9.0)]
***C***_**13**_	[(6.5,8.2),9.3,(9.7,10.0)]	[(6.5,8.2),9.3,(9.7,10.0)]	[(6.2,7.5),8.7,(9.2,9.8)]	[(4.8,6.2),7.7,(8.5,9.7)]	[(4.5,6.2),7.7,(8.5,9.2)]
***C***_**14**_	[(7.5,8.8),9.7,(9.8,10.0)]	[(6.5,8.2),9.3,(9.7,10.0)]	[(5.2,6.8),8.3,(9.0,9.8)]	[(6.5,8.2),9.3,(9.7,10.0)]	[(7.5,8.8),9.7,(9.8,10.0)]
***C***_**15**_	[(5.2,6.8),8.3,(9.0,9.8)]	[(4.2,5.5),7.0,(8.0,9.0)]	[(6.5,8.2),9.3,(9.7,10.0)]	[(6.5,8.2),9.3,(9.7,10.0)]	[(6.5,8.2),9.3,(9.7,10.0)]
***C***_**16**_	[(6.5,8.2),9.3,(9.7,10.0)]	[(7.5,8.8),9.7,(9.8,10.0)]	[(7.5,8.8),9.7,(9.8,10.0)]	[(6.2,7.5),8.7,(9.2,9.8)]	[(4.8,6.2),7.7,(8.5,9.7)]
***C***_**17**_	[(4.5,6.2),7.7,(8.5,9.2)]	[(3.8,4.8),6.3,(7.5,8.8)]	[(4.2,5.5),7.0,(8.0,9.0)]	[(5.2,6.8),8.3,(9.0,9.8)]	[(4.2,5.5),7.0,(8.0,9.0)]
***C***_**18**_	[(2.3,3.5),5.0,(6.3,7.5)]	[(0.8,2.2),3.7,(5.2,6.2)]	[(0.0,1.5),3.0,(4.5,5.5)]	[(1.7,2.8),4.3,(5.8,6.8)]	[(3.8,4.8),6.3,(7.5,8.8)]
***C***_**19**_	[(3.8,4.8),6.3,(7.5,8.8)]	[(2.3,3.5),5.0,(6.3,7.5)]	[(3.5,4.8),6.3,(7.5,8.3)]	[(3.8,4.8),6.3,(7.5,8.8)]	[(4.2,5.5),7.0,(8.0,9.0)]
***C***_**20**_	[(3.8,4.8),6.3,(7.5,8.8)]	[(2.3,3.5),5.0,(6.3,7.5)]	[(4.2,5.5),7.0,(8.0,9.0)]	[(3.3,4.8),6.3,(7.3,8.3)]	[(3.2,4.2),5.7,(7.0,8.2)]
***C***_**21**_	[(4.8,6.2),7.7,(8.5,9.7)]	[(4.8,6.2),7.7,(8.5,9.7)]	[(4.2,5.5),7.0,(8.0,9.0)]	[(3.2,4.2),5.7,(7.0,8.2)]	[(4.8,6.2),7.7,(8.5,9.7)]
***C***_**22**_	[(4.8,6.2),7.7,(8.5,9.7)]	[(3.8,4.8),6.3,(7.5,8.8)]	[(5.2,6.8),8.3,(9.0,9.8)]	[(3.2,4.2),5.7,(7.0,8.2)]	[(5.5,7.5),9.0,(9.5,10.0)]

In this stage, as the third step, all values of every alternative are converted into a comparability sequence for preventing some attributes being neglected. For proper conducting of these processes, the *IVFNs of the aggregated decision matrix for the rating of alternatives* should be defuzzified based on Eq (4) as shown in Table 7.

**Table 7:** Defuzzified decision matrix for rating of alternatives.

	*A*_1_	*A*_2_	*A*_3_	*A*_4_	*A*_5_
***C***_**1**_	8.42	8.06	8.42	7.58	8.06
***C***_**2**_	6.34	6.34	5.69	4.64	3.46
***C***_**3**_	7.44	8.91	7.44	6.34	7.44
***C***_**4**_	8.42	8.42	8.91	6.96	7.79
***C***_**5**_	9.29	9.29	8.55	8.42	8.42
***C***_**6**_	9.29	8.91	8.91	8.06	8.55
***C***_**7**_	8.91	8.91	8.06	8.91	8.91
***C***_**8**_	8.06	8.42	6.96	6.49	8.06
***C***_**9**_	6.96	5.23	6.96	8.91	8.42
***C***_**10**_	8.06	8.42	6.96	6.96	8.06
***C***_**11**_	6.96	8.91	7.79	8.42	7.44
***C***_**12**_	8.91	6.96	6.34	8.06	6.96
***C***_**13**_	8.91	8.91	8.42	7.58	7.44
***C***_**14**_	9.29	8.91	8.06	8.91	9.29
***C***_**15**_	8.06	6.96	8.91	8.91	8.91
***C***_**16**_	8.91	9.29	9.29	8.42	7.58
***C***_**17**_	7.44	6.49	6.96	8.06	6.96
***C***_**18**_	5.23	4.02	3.43	4.64	6.49
***C***_**19**_	6.49	5.23	6.34	6.49	6.96
***C***_**20**_	6.49	5.23	6.96	6.30	5.88
***C***_**21**_	7.58	7.58	6.96	5.88	7.58
***C***_**22**_	7.58	6.49	8.06	5.88	8.55

### Evaluating airline service quality

#### Step 3: Calculating the comparative series for each criterion

Based on Table 7, the comparability sequence for each criterion can be calculated as shown in Table 8.

**Table 8:** Comparability sequence for each criterion.

	*A*_1_	*A*_2_	*A*_3_	*A*_4_	*A*_5_
***C***_**1**_	1.00	0.58	1.00	0.00	0.58
***C***_**2**_	1.00	1.00	0.77	0.41	0.00
***C***_**3**_	0.43	1.00	0.43	0.00	0.43
***C***_**4**_	0.75	0.75	1.00	0.00	0.43
***C***_**5**_	1.00	1.00	0.15	0.00	0.00
***C***_**6**_	1.00	0.69	0.69	0.00	0.40
***C***_**7**_	1.00	1.00	0.00	1.00	1.00
***C***_**8**_	0.82	1.00	0.24	0.00	0.82
***C***_**9**_	0.47	0.00	0.47	1.00	0.87
***C***_**10**_	0.76	1.00	0.00	0.00	0.76
***C***_**11**_	0.00	1.00	0.43	0.75	0.25
***C***_**12**_	1.00	0.24	0.00	0.67	0.24
***C***_**13**_	1.00	1.00	0.66	0.09	0.00
***C***_**14**_	1.00	0.69	0.00	0.69	1.00
***C***_**15**_	0.56	0.00	1.00	1.00	1.00
***C***_**16**_	0.78	1.00	1.00	0.49	0.00
***C***_**17**_	0.60	0.00	0.30	1.00	0.30
***C***_**18**_	0.59	0.19	0.00	0.39	1.00
***C***_**19**_	0.73	0.00	0.64	0.73	1.00
***C***_**20**_	0.73	0.00	1.00	0.62	0.38
***C***_**21**_	1.00	1.00	0.64	0.00	1.00
***C***_**22**_	0.64	0.23	0.82	0.00	1.00

#### Step 4. Determining the weights of criteria

First, subjective weights of criteria based on the averaging score method are calucalted. After calculating the comparative series for each criterion, the weights of criteria can be determined by combining subjective weights based on the *averaging score method* and objective weights derived from entropy measure.

First, for deriving subjective weights of criteria, importance weights of criteria as assessed by 3 experts were aggregated using the *averaging score method* as shown in Table 9. For example, according to Eqs (13) and (14), the aggregated IFVNs for the importance weights of the 1st criteria could be calculated as

$$\begin{array}{l} C_{1}=[(\frac{0.45+0.25+0}{3},\frac{0.55+0.35+0.15}{3}),\frac{0.7+0.5+0.3}{3},(\frac{0.8+0.65+0.45}{3},\frac{0.95+0.75+0.55}{3})]\\ =[(0.23,0.35),0.5,(0.63,0.75)]. \end{array}$$

**Table 9:** Subjective weights based on the averaging score method.

	Weights assessed by 3 decision makers (*DM*_1_,*DM*_2_,*DM*_3_)	Aggregated IFVNs for weights of criteria	Defuzzified subjective weights of criteria ($W_{j}^{Sub}$)
***C***_1_	(MH,M,ML)	[(0.23,0.35),0.50,(0.63,0.75)]	0.0294
***C***_2_	(H,H,MH)	[(0.52,0.68),0.83,(0.90,0.98)]	0.0454
***C***_3_	(MH,MH,M)	[(0.38,0.48),0.63,(0.75,0.88)]	0.0365
***C***_4_	(H,MH,VH)	[(0.62,0.75),0.87,(0.92,0.98)]	0.0474
***C***_5_	(H,H,VH)	[(0.65,0.82),0.93,(0.97,1.00)]	0.0502
***C***_6_	(H,H,MH)	[(0.52,0.68),0.83,(0.90,0.98)]	0.0454
***C***_7_	(H,VH,H)	[(0.65,0.82),0.93,(0.97,1.00)]	0.0502
***C***_8_	(MH,VH,VH)	[(0.72,0.82),0.90,(0.93,0.98)]	0.0495
***C***_9_	(VH,VH,H)	[(0.75,0.88),0.97,(0.98,1.00)]	0.0523
***C***_10_	(H,H,MH)	[(0.52,0.68),0.83,(0.90,0.98)]	0.0454
***C***_11_	(MH,H,H)	[(0.52,0.68),0.83,(0.90,0.98)]	0.0454
***C***_12_	(VH,H,VH)	[(0.75,0.88),0.97,(0.98,1.00)]	0.0523
***C***_13_	(MH,VH,VH)	[(0.72,0.82),0.90,(0.93,0.98)]	0.0495
***C***_14_	(VH,VH,VH)	[(0.85,0.95),1.00,(1.00,1.00)]	0.0545
***C***_15_	(H,H,MH)	[(0.52,0.68),0.83,(0.90,0.98)]	0.0454
***C***_16_	(VH,VH,H)	[(0.75,0.88),0.97,(0.98,1.00)]	0.0523
***C***_17_	(MH,VH,H)	[(0.62,0.75),0.87,(0.92,0.98)]	0.0474
***C***_18_	(ML,M,MH)	[(0.23,0.35),0.50,(0.63,0.75)]	0.0294
***C***_19_	(MH,MH,H)	[(0.48,0.62),0.77,(0.85,0.97)]	0.0427
***C***_20_	(H,H,MH)	[(0.52,0.68),0.83,(0.90,0.98)]	0.0454
***C***_21_	(MH,H,VH)	[(0.62,0.75),0.87,(0.92,0.98)]	0.0474
***C***_22_	(M,MH,MH)	[(0.38,0.48),0.63,(0.75,0.88)]	0.0365

Based on the aggregated IFVNs for weights of criteria, the defuzzified values of the subjective weights of criteria can be calculated using Eqs (15) and (16) as shown in Table 9. Here, the defuzzified values of the subjective weights are normalized to satisfy the condition

$$\sum_{j=1}^{n}W_{j}^{Sub}=1.$$ (25)

Second, objective weights of criteria using entropy measure are calculated. According to Eqs (17), (18), (19) and (20), we can calculate *e*_*j*_, *div*_*i*_, and $W_{j}^{Sub}$ respectively as shown in Table 10.

**Table 10:** Objective weights based on entropy measure.

	Entropy value (*e*_*j*_)	Degree of diversification (*div*_*i*_)	Objective weights ($W_{j}^{Obj}$)
***C***_**1**_	0.9995	0.0005	0.0055
***C***_**2**_	0.9856	0.0144	0.1747
***C***_**3**_	0.9964	0.0036	0.0441
***C***_**4**_	0.9978	0.0022	0.0265
***C***_**5**_	0.9993	0.0007	0.0081
***C***_**6**_	0.9993	0.0007	0.0085
***C***_**7**_	0.9995	0.0005	0.0058
***C***_**8**_	0.9970	0.0030	0.0364
***C***_**9**_	0.9899	0.0101	0.1220
***C***_**10**_	0.9980	0.0020	0.0241
***C***_**11**_	0.9976	0.0024	0.0289
***C***_**12**_	0.9953	0.0047	0.0565
***C***_**13**_	0.9982	0.0018	0.0224
***C***_**14**_	0.9992	0.0008	0.0098
***C***_**15**_	0.9973	0.0027	0.0331
***C***_**16**_	0.9983	0.0017	0.0211
***C***_**17**_	0.9983	0.0017	0.0206
***C***_**18**_	0.9851	0.0149	0.1807
***C***_**19**_	0.9973	0.0027	0.0325
***C***_**20**_	0.9972	0.0028	0.0343
***C***_**21**_	0.9972	0.0028	0.0340
***C***_**22**_	0.9942	0.0058	0.0703

Lastly, based on the objective and subjective weights, the integrated weights of the criteria can be calculated using Eq (21) as shown in Table 11. In this case, we set ***α*** = 0.5 to reflect the objective and subjective weights equally.

**Table 11:** Integrated weights of criteria.

	Subjective weights ($W_{j}^{Sub}$)	Objective weights ($W_{j}^{Obj}$)	Integrated weights ($W_{j}^{Integ}$)
***C***_**1**_	0.0294	0.0055	0.0175
***C***_**2**_	0.0454	0.1747	0.1101
***C***_**3**_	0.0365	0.0441	0.0403
***C***_**4**_	0.0474	0.0265	0.0369
***C***_**5**_	0.0502	0.0081	0.0291
***C***_**6**_	0.0454	0.0085	0.0270
***C***_**7**_	0.0502	0.0058	0.0280
***C***_**8**_	0.0495	0.0364	0.0430
***C***_**9**_	0.0523	0.1220	0.0872
***C***_**10**_	0.0454	0.0241	0.0347
***C***_**11**_	0.0454	0.0289	0.0372
***C***_**12**_	0.0523	0.0565	0.0544
***C***_**13**_	0.0495	0.0224	0.0360
***C***_**14**_	0.0545	0.0098	0.0321
***C***_**15**_	0.0454	0.0331	0.0393
***C***_**16**_	0.0523	0.0211	0.0367
***C***_**17**_	0.0474	0.0206	0.0340
***C***_**18**_	0.0294	0.1807	0.1051
***C***_**19**_	0.0427	0.0325	0.0376
***C***_**20**_	0.0454	0.0343	0.0399
***C***_**21**_	0.0474	0.0340	0.0407
***C***_**22**_	0.0365	0.0703	0.0534

#### Step 5–6: Setting the standard series (reference sequence) and calculating the overall grey relational coefficients

Working with the reference sequence ***X***_0_ as (***x***_01_,***x***_02_,…,***x***_0***j***_,…,***x***_022_) = (1,1,…,1,…,1), the overall grey relational coefficients for each dimension are calculated using Eqs (22) and (23) as shown in Table 12. In this case, we set ***ζ*** = 0.5.

**Table 12:** Overall grey relational coefficients.

	*A*_1_	*A*_2_	*A*_3_	*A*_4_	*A*_5_
***C***_**1**_	0.3333	0.4649	0.3333	1.0000	0.4649
***C***_**2**_	0.3333	0.3333	0.3930	0.5508	1.0000
***C***_**3**_	0.5397	0.3333	0.5397	1.0000	0.5397
***C***_**4**_	0.4010	0.4010	0.3333	1.0000	0.5405
***C***_**5**_	0.3333	0.3333	0.7664	1.0000	1.0000
***C***_**6**_	0.3333	0.4193	0.4193	1.0000	0.5566
***C***_**7**_	0.3333	0.3333	1.0000	0.3333	0.3333
***C***_**8**_	0.3801	0.3333	0.6718	1.0000	0.3801
***C***_**9**_	0.5156	1.0000	0.5156	0.3333	0.3661
***C***_**10**_	0.3982	0.3333	1.0000	1.0000	0.3982
***C***_**11**_	1.0000	0.3333	0.5405	0.4010	0.6703
***C***_**12**_	0.3333	0.6762	1.0000	0.4278	0.6762
***C***_**13**_	0.3333	0.3333	0.4294	0.8412	1.0000
***C***_**14**_	0.3333	0.4193	1.0000	0.4193	0.3333
***C***_**15**_	0.4697	1.0000	0.3333	0.3333	0.3333
***C***_**16**_	0.3908	0.3333	0.3333	0.5048	1.0000
***C***_**17**_	0.4526	1.0000	0.6253	0.3333	0.6253
***C***_**18**_	0.4596	0.7224	1.0000	0.5591	0.3333
***C***_**19**_	0.4073	1.0000	0.4368	0.4073	0.3333
***C***_**20**_	0.4073	1.0000	0.3333	0.4476	0.5714
***C***_**21**_	0.3333	0.3333	0.4402	1.0000	0.3333
***C***_**22**_	0.4400	0.6867	0.3797	1.0000	0.3333

#### Step 7: Calculating the overall grey relational grade

After calculating the overall grey relational coefficients, the overall grey relational grade is derived by applying the weighted average of each grey relational grade. Based on the integrated weights of criteria (see Table 11) and the overall grey relational coefficients in Table 12, the grey relational grades are calculated, finally, as shown in Table 13.

**Table 13:** Overall grey relational grades and ranking.

	*A*_1_	*A*_2_	*A*_3_	*A*_4_	*A*_5_
***C***_**1**_	0.0058	0.0081	0.0058	0.0175	0.0081
***C***_**2**_	0.0367	0.0367	0.0432	0.0606	0.1101
***C***_**3**_	0.0218	0.0134	0.0218	0.0403	0.0218
***C***_**4**_	0.0148	0.0148	0.0123	0.0369	0.0200
***C***_**5**_	0.0097	0.0097	0.0223	0.0291	0.0291
***C***_**6**_	0.0090	0.0113	0.0113	0.0270	0.0150
***C***_**7**_	0.0093	0.0093	0.0280	0.0093	0.0093
***C***_**8**_	0.0163	0.0143	0.0289	0.0430	0.0163
***C***_**9**_	0.0449	0.0872	0.0449	0.0291	0.0319
***C***_**10**_	0.0138	0.0116	0.0347	0.0347	0.0138
***C***_**11**_	0.0372	0.0124	0.0201	0.0149	0.0249
***C***_**12**_	0.0181	0.0368	0.0544	0.0233	0.0368
***C***_**13**_	0.0120	0.0120	0.0154	0.0303	0.0360
***C***_**14**_	0.0107	0.0135	0.0321	0.0135	0.0107
***C***_**15**_	0.0184	0.0393	0.0131	0.0131	0.0131
***C***_**16**_	0.0144	0.0122	0.0122	0.0185	0.0367
***C***_**17**_	0.0154	0.0340	0.0213	0.0113	0.0213
***C***_**18**_	0.0483	0.0759	0.1051	0.0587	0.0350
***C***_**19**_	0.0153	0.0376	0.0164	0.0153	0.0125
***C***_**20**_	0.0162	0.0399	0.0133	0.0178	0.0228
***C***_**21**_	0.0136	0.0136	0.0179	0.0407	0.0136
***C***_**22**_	0.0235	0.0367	0.0203	0.0534	0.0178
**Grey relational grade**	0.4253	0.5802	0.5949	0.6384	0.5566
**Ranking**	**5**	**3**	**2**	**1**	**4**

As a result, the ranking of the five airline services is ***A***_4_ > ***A***_3_ > ***A***_2_ > ***A***_5_ > ***A***_1_. In this case, ***A***_4_ is the best choice. Accordingly, ***A***_4_ has the high service quality considering the various criteria, and it can be the benchmarking airline service when other airline services want to improve their service quality.

In detail, ***A***_4_ was the best alternative based on consideration of the various criteria. This was due to the fact that the grey relational grades of many criteria in ***A***_4_ were the best among the various alternatives. Specifically, the evaluation scores of the nine criteria including up-to-date equipment & technology (***C***_1_), appearance of the physical facilities of this airline (***C***_3_), appearance of flight attendants (***C***_4_), courtesy of flight attendants (***C***_5_), handling of delays (***C***_6_), flight attendants’ approach to unexpected situations (***C***_8_), flight safety (***C***_10_), convenient ticketing process (***C***_21_), and customer complaint handling (***C***_22_) were the highest among the alternatives. This means that the comparability sequence of ***A***_4_ was the most similar to the reference sequence.

### Comprehensive discussion of results

The five airline service alternatives were evaluated by application of the proposed approach. To prove the full potential of the proposed approach, three issues regarding the proposed approach should be discussed. First, because an important issue of airline service quality evaluation is to know the most influential main criteria affecting evaluation results, a detailed investigation of integrated weights and subjective/objective weights should be carried out. Second, a sensitivity analysis should be conducted to investigate subjective and objective weights’ respective influence levels in the evaluation problem. Lastly, validation of results by comparing the results with other representative MCDM methods should be progressed.

#### Detailed investigation for weights of criteria

As integrated weights are calculated based on a combination of subjective and objective weights, the distribution between those subjective and objective weights can affect integrated weight values. Table 10 provides the integrated weight values obtained in the present study. We can know that comfort and cleanliness of seat (***C***_2_) in the tangibles dimension, flight attendants’ behavior toward delayed passengers (***C***_18_) in the empathy dimension, and flight attendants’ willingness to help (***C***_9_) in the responsiveness dimension are important criteria in the evaluation of airline service quality. Meanwhile, we also know that up-to-date equipment & technology (***C***_1_) is relatively less important.

In terms of the gap between subjective and objective weights, that for flight attendants’ behavior toward delayed passengers (***C***_18_) is the largest. This means that ***C***_18_ is assessed as high importance in terms of objective weights, whereas the subjective preferences of decision makers towards ***C***_18_ are low. Meanwhile, courtesy of flight attendants (***C***_5_) shows, among the 22 criteria, the smallest gap between subjective and objective weights. This implies that there is no difference between subjective preference and objective importance weights derived from the decision matrix.

#### Sensitivity analysis

**Fig 3:**
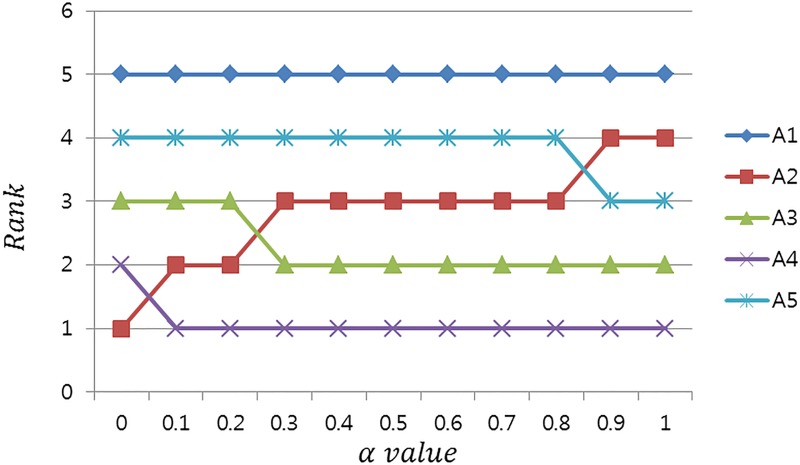
Results of sensitivity analysis.

A sensitivity analysis also was conducted to investigate the influence levels of subjective and objective weights. The aim of sensitivity analysis is to observe the ranking order when the coefficient of subjective weights changes. The results of the sensitivity analysis are plotted in Fig 3.

The rankings of ***A***_4_ were not at all affected by the *α* value except when *α* = 0. This means that airline service alternative ***A***_4_ provides high service quality considering both subjective and objective weights. Additionally, the rankings of ***A***_1_ also were not at all affected by the *α* value. On the other hand, the rankings of ***A***_2_ were improved as the *α* value decreased. This fact reveals that ***A***_2_ has higher service quality when one focuses on objective weights. Also, the rankings of ***A***_3_ and ***A***_4_ were high when the *α* value was high, indicating that their rank order were increased when the influence of subjective weights was increased. In other words, they scored higher service quality levels when subjective weights assessed by experts were considered to be important.

#### Validation test for comparing the results with other methods

This study also conducted validation test for demonstrating in order to effectively demonstrate the methods improvement over current studies by comparing the results with other MCDM methods utilized in current studies. In previous studies, TOPSIS has been well applied for evaluating business competition [63] or service quality [3] in airline industry. In addition, in recent work, Liou, Tsai (1) applied the VIKOR method to improve the service qualities of domestic Taiwanese airlines.

We compared the results of proposed approach with those of TOPSIS and VIKOR which are MCDM methods well utilized in current studies. For the validation, importance weights of criteria derived from integrated weight approach is applied to other MCDM methods. Here, we set *α* = 0.5 to reflect the objective and subjective weights equally.

Table 14 represents that the results of the proposed approach are similar to those of TOPSIS which is representative MCDM methods. The results of upper ranking group (***A***_2_, ***A***_3_, ***A***_4_) and lower ranking group (***A***_1_, ***A***_5_) were same in TOPSIS methods. Thus, even though GRA follows a simple and easy calculation process and there are uncertainty, multi-input, and data incompleteness in the evaluation environment, it provides the precise processing results. Especially, in uncertainty-intensive environments such as the airline service industry, GRA has competitive advantages.

In addition, the results of proposed approach and TOPSIS were different with those of VIKOR. Actually VIKOR method provides a maximum ‘‘group utility of majority” and a minimum ‘‘individual regret of opponent (consideration of dissatisfaction)”, so decision makers can determine compromise solutions based on their negotiated preferences. In other words, where unsatisfactory attributes can remarkably affect the selection of an entire service, VIKOR, compared with other MCDM methods. However, in the evaluation of airline service quality, achieving the desired quality level is more important than consideration for dissatisfaction. This is because there are a lot of dimension and sub-criteria in the evaluation of airline service quality, so it is more effective to improve service quality by focusing more on group utility of majority than on individual regret of opponent. Thus, it is reasonable to apply GRA which is utilized in proposed approach rather than VIKOR in this situation. In summary, the proposed approach is better and reasonable than TOPSIS and VIKOR, which were used in previous studies.

**Table 14:** Comparison with other MCDM methods in current studies.

MCDM	Ordering
Proposed approach (In case of *α* = 0.5)	A4 > A3 > A2 > A5 > A1
TOPSIS [3, 63]	A3 > A4 > A2 > A5 > A1
VIKOR [1]	A1 > A2 > A4 > A5 > A3

## Conclusion

This paper developed an interval-valued fuzzy GRA with SERVPERF based on both subjective and objective weights for evaluation of airline service quality. The proposed approach consists of two main stages: *defining the problem situation* using SERVPERF and interval-valued fuzzy sets, and *evaluating the airline service quality* using interval-valued fuzzy GRA and integrated weights. In the initial, *defining the problem situation* stage, first, several airline services are selected as the evaluation alternatives. Then, for those airline services, a decision matrix is established on the basis of SERVPERF in order to obtain assessment scores for interval-valued fuzzy sets. In the subsequent, *evaluating the airline service quality* stage, comparative series for each criterion are first calculated. In this step, to calculate the comparative series, the *IVFNs of the aggregated decision matrix for the rating of alternatives* should be defuzzified. As the next step, the integrated weights of criteria are calculated by combining the subjective and objective weights. In this step, the averaging score method is utilized to obtain the subjective preferences of the different experts and the entropy measure is applied to derive the objective weights. Then, based on the reference sequence, the overall grey relational coefficients for each dimension are calculated. Finally, the grey relational grades can be calculated using the overall grey relational coefficients and integrated weights of criteria.

The contribution and potential utility of the proposed approach can be explained by three. First, it reflects the various characteristics of airline service by utilizing SERVPERF, which incorporating five dimensions and 22 criteria to represent airline service characteristics. This study addresses the limitations of the previous, SERVQUAL approach that focuses only on the gap model in which service quality is a function of the difference between the perceptions and expectations of a service. We utilized not the gap measure but rather the performance-based measure of service quality in order to more effectively reflect airline service characteristics. Also, the SERVPERF criteria, included in this paper, are by no means fixed, but can be customized according to the judgment of a firm. Second, from the methodological perspective, this paper also contributes to the field in that it proposes interval-valued fuzzy GRA and integrated weights of criteria. Advanced fuzzy logics are applied in the GRA method to effectively cover the uncertainty and the vagueness in airline service evaluations. It can provide the direction of further studies focusing on the evaluation of airline service in the fuzzy environment. Additionally, in order to utilize interval-valued fuzzy GRA within the unique context of airline service evaluation, a novel weighting technique is proposed combining the subjective weighting method (i.e., the averaging score method) and the objective weighting method (i.e., entropy weights) to integrate the subjective preferences of decision makers with decision-matrix-derived objective information.

Notwithstanding these several contributions of this paper, it also has some limitations that provide paths for future research. First, the information-aggregation stage of the proposed approach needs to be made more effective, which goal might be achieved by development of advanced aggregation operators. Second, the approach requires further validation, specifically by application of other objective and subjective weighting methods. Third, other advanced fuzzy logics such as Pythagorean fuzzy sets can be applied to handle more uncertainty and the effectiveness of various fuzzy logics can be compared and verified. Lastly, the type of case study should be conducted also for additional airline services so as to make possible the development of a more concrete framework.
