# Correction: Development of interval-valued fuzzy GRA with SERVPERF based on subjective and objective weights for evaluation of airline service quality: A case study of Korea low-cost carriers

**DOI:** 10.1371/journal.pone.0222541

**Published:** 2019-09-11

**Authors:** The PLOS ONE Staff

The following information is missing from the Funding statement: This work was supported by the National Research Foundation of Korea (NRF) grant funded by the Korea government (MSIT) (2019R1F1A1058719 and 2019R1G1A1006073). The publisher apologizes for the error.
